# Computed Tomography and Magnetic Resonance Imaging Features of the Temporomandibular Joint in Two Normal Camels

**DOI:** 10.1155/2012/242065

**Published:** 2011-10-20

**Authors:** Alberto Arencibia, Diego Blanco, Nelson González, Miguel A. Rivero

**Affiliations:** Department of Morphology, Veterinary Faculty, University of Las Palmas de Gran Canaria, Trasmontaña s/n, Gran Canaria, 35413 Las Palmas, Spain

## Abstract

Computed tomography (CT) and magnetic resonance (MR) image features of the temporomandibular joint (TMJ) and associated structures in two mature dromedary camels were obtained with a third-generation equipment CT and a superconducting magnet RM at 1.5 Tesla. Images were acquired in sagittal and transverse planes. Medical imaging processing with imaging software was applied to obtain postprocessing CT and MR images. Relevant anatomic structures were identified and labelled. The resulting images provided excellent anatomic detail of the TMJ and associated structures. Annotated CT and MR images from this study are intended as an anatomical reference useful in the interpretation for clinical CT and MR imaging studies of the TMJ of the dromedary camels.

## 1. Introduction

The temporomandibular joint (TMJ) is a synovial condylar joint between the base of the zygomatic process of the temporal bone and the condylar process of the mandible; its main articular components are the synovial pouches, articular disc, caudal and lateral ligaments, and joint capsule [1]. 

In veterinary medicine, the exploration of the anatomical structures located in the TMJ and the evaluation of the soft tissues turn out to be laborious due to its complex anatomical organization [2–5], which makes it difficult to diagnose morphological alterations by means of physical exploration and conventional radiographic studies [6–8]. 

Nowadays, modern image-based diagnostic techniques, especially computed tomography (CT) [9–14], and magnetic resonance imaging (MRI) [15–21], make possible to obtain body sections from different tomographic planes, achieving images with a good anatomical resolution, high contrast between different structures, and excellent tissue-like differentiation. The applications of CT and MRI have revolutionized the practice of veterinary diagnostic imaging. In large animals, several studies have demonstrated the clinical value of CT and MRI of the TMJ [22–25]. To the author's knowledge, there is no published material describing the results of CT and MRI of the mature camel TMJ. An accurate interpretation of the CT and MRI normal anatomy is necessary for the evaluation of pathological tissues.

The objective of this study was to provide an overview of the normal anatomy of the TMJ of the dromedary camel using CT, MR images, and transverse gross anatomical section.

## 2. Methods

### 2.1. Animals

Two male mature dromedary camels' cadaver heads were used for this study. One was from a 4-year-old (525 kg bodyweight) and the other from an 8-year-old (638 kg bodyweight) one. Camels were dead for medical reasons unrelated to the TMJ diseases and did not show lesions in the head that could influence our results. 

The camels belonged to a Camel Farm located in Fataga, Gran Canaria, Canary Island, Spain. Both heads, sectioned at the level of atlantoaxial joint, were refrigerated and imaged immediately to minimize postmortem changes.

### 2.2. CT Scan Technique

CT imaging was performed at the Radiodiagnostic Service of the Hospital Universitario Insular of Las Palmas de Gran Canaria (Spain), with a Toshiba 600 HQ scanner (third-generation equipment, Toshiba Medical Imaging System, Tustin, Calif, USA). Throughout the procedure, the heads were positioned in ventral recumbency during scanning time. 

CT images from the TMJ were obtained in sagittal and transverse planes with 120 kV and 130 Ma. Best image quality was obtained by adjusting the window widths (WWs) and window levels (WLs) setting. 

For visualizing soft tissue structures, a soft-tissue window setting (WW = 4000; WL = 335) was used. In addition, medical imaging processing software (Osirix) was applied to obtain postprocessing sagittal and transverse CT images with clut VR muscles-bones.

### 2.3. MRI Technique

MR imaging was performed at the Radiodiagnostic Service of the Clinica San Roque of Las Palmas de Gran Canaria (Spain), using a superconducting magnet operating at field strength of 1.5 Tesla (Genesis Sigma; General Electric Medical System, USA) and a human coil. Images were acquired in sagittal and transverse planes with fast spin-echo (FSE) sequences. Throughout the procedure, the heads were positioned in ventral recumbency during scanning time. 

FSE T1-weighted transverse MR images were obtained with the following parameters: repetition time (TR) = 340 ms, echo time (TE) = 8 ms, 256 × 224 matrix, and one excitation. For FSE T2-weighted transverse images, the TR was 6400 ms, TE was 105 ms, 256 × 224 matrix, and one excitation. In addition, medical imaging processing software (Osirix) was applied to obtain postprocessing T1- and T2-weighted MR images with clut VR muscles-bones.

### 2.4. Anatomical Evaluation

CT and MR images were compared to transverse anatomical section and with anatomy books [1, 26–28], to identify the normal CT and MRI anatomy of the TMJ of the dromedary camel. Clinically, several structures of the TMJ were identified and labelled according to an internationally accepted veterinary anatomical nomenclature [27].

## 3. Results

**Figure 1:**
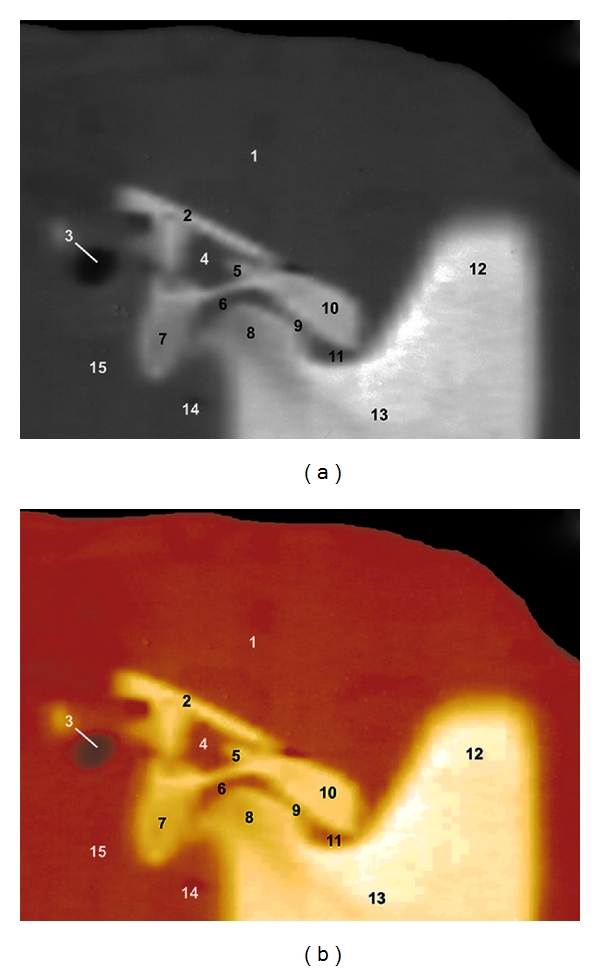
Sagittal CT images at the level of the TMJ in a mature camel. (a) Soft tissue window CT image (WW = 4000; WL = 335) and (b) postprocessing VR muscles-bones CT image (Osirix imaging software). Right lateral view. 1 is temporal muscle; 2 is border of the temporal fossa; 3 is external acoustic meatus; 4 is temporal venous sinus; 5 is zygomatic process of the temporal bone; 6 is articular disc; 7 is retroarticular process; 8 is mandibular condyle; 9 is joint capsule; 10 is articular tubercle; 11 is mandibular notch; 12 is coronoid process of mandible; 13 is ramus of mandible; 14 is digastric muscle; 15 is parotid salivary gland.

**Figure 2:**
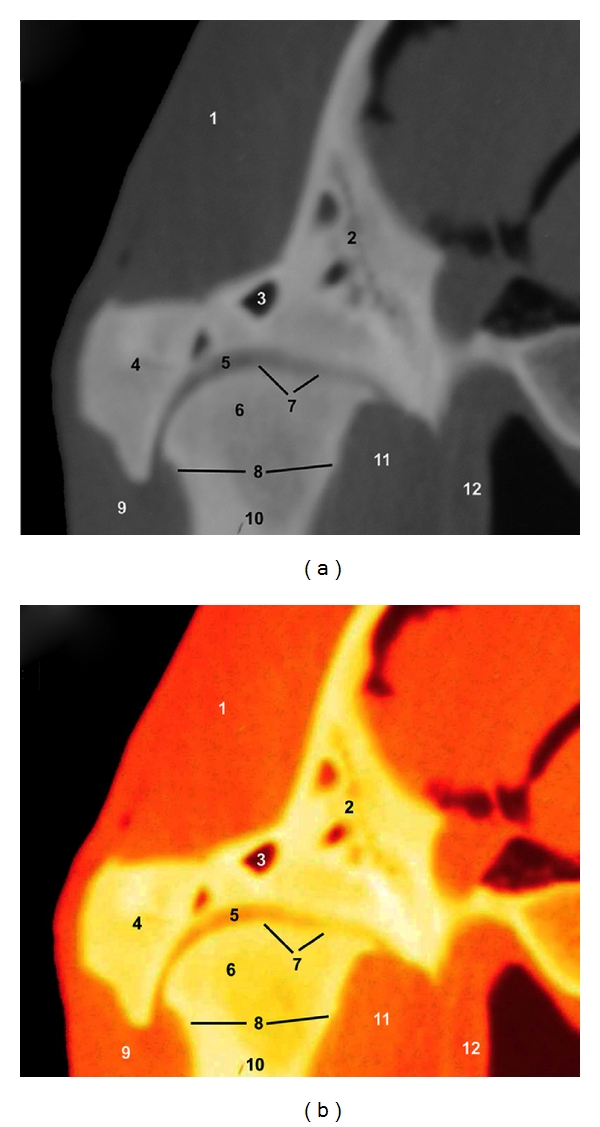
Transverse CT images at the level of the TMJ in a mature camel. (a) Soft tissue window CT image (WW = 4000; WL = 335) and (b) postprocessing VR muscles-bones CT image (Osirix imaging software). Caudal view. 1 is temporal muscle; 2 is squamous part of the temporal bone; 3 is temporal venous sinus; 4 is zygomatic process of the temporal bone; 5 is articular disc; 6 is mandibular condyle; 7 is joint capsule; 8 is cortical bone; 9 is masseter muscle; 10 is ramus of mandible; 11 is lateral pterygoid muscle; 12 is tensor and levator veli palatini muscles.

**Figure 3:**
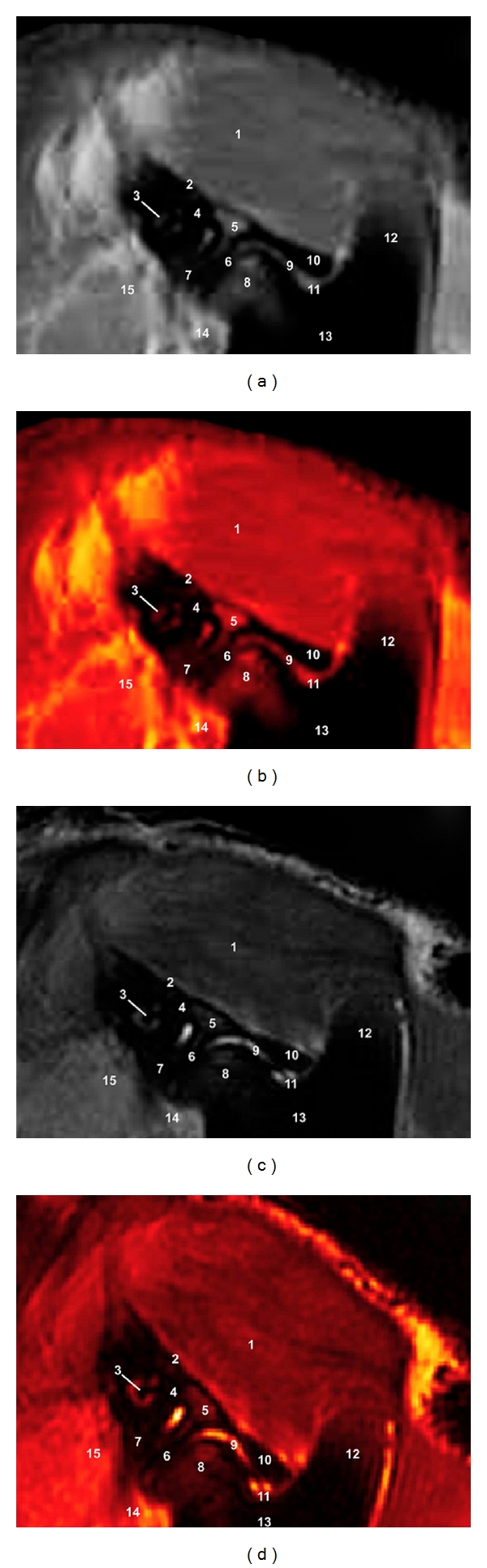
Sagittal FSE MR images at the level of the TMJ in a mature camel. (a) T1-weighted MR image, (b) postprocessing VR muscles-bones T1-weighted MR image (Osirix imaging software), (c) T2-weighted MR image, and (d) postprocessing VR muscles-bones T2-weighted MR image (Osirix imaging software). Right lateral view. 1 is temporal muscle; 2 is border of the temporal fossa; 3 is external acoustic meatus; 4 is temporal venous sinus; 5 is zygomatic process of the temporal bone; 6 is articular disc; 7 is retroarticular process; 8 is mandibular condyle; 9 is joint capsule; 10 is articular tubercle; 11 is mandibular notch; 12 is coronoid process of mandible; 13 is ramus of mandible; 14 is digastric muscle; 15 is parotid salivary gland.

**Figure 4:**
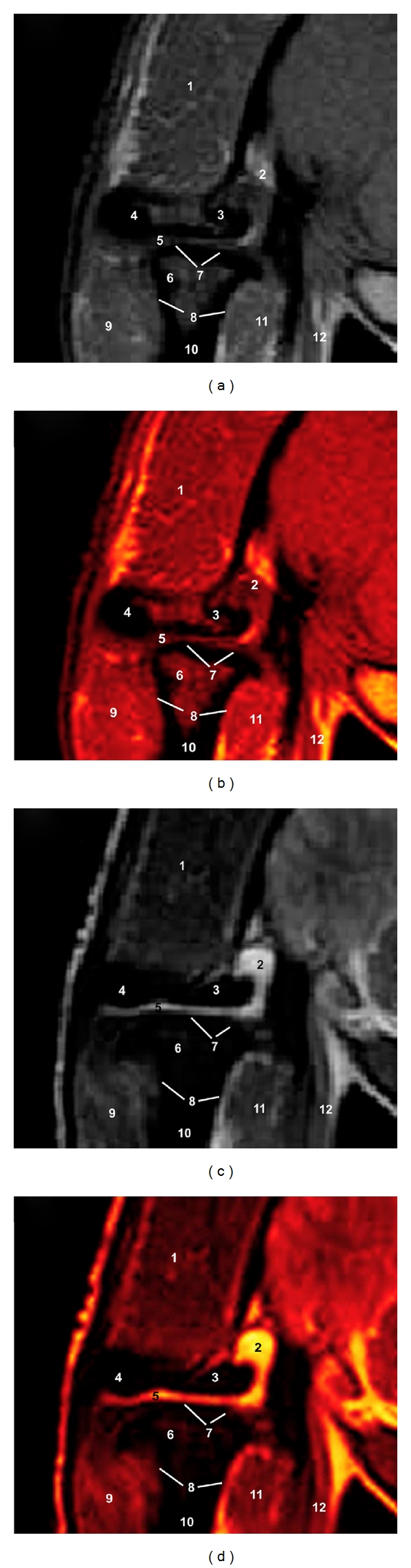
Transverse FSE MR images at the level of the left TMJ in a mature camel. (a) T1-weighted MR image, (b) postprocessing VR muscles-bones T1-weighted MR image (Osirix imaging software), (c) T2-weighted MR image and (d) postprocessing VR muscles-bones T2-weighted MR image (Osirix imaging software). Caudal view. 1 is temporal muscle; 2 is squamous part of the temporal bone; 3 is temporal venous sinus; 4 is zygomatic process of the temporal bone; 5 is articular disc; 6 is mandibular condyle; 7 is joint capsule; 8 is cortical bone; 9 is masseter muscle; 10 is ramus of mandible; 11 is lateral pterygoid muscle; 12 is tensor and levator veli palatini muscles.

**Figure 5:**
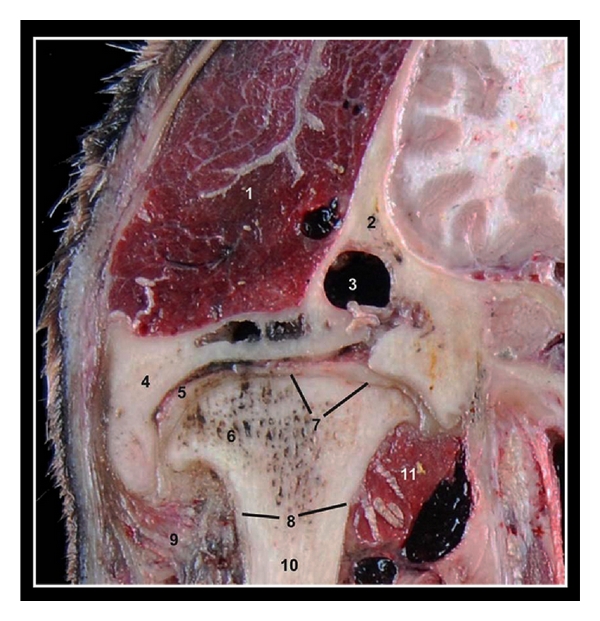
Transverse anatomical section at the level of the left TMJ in a mature camel. Caudal view. 1 is temporal muscle; 2 is squamous part of the temporal bone; 3 is temporal venous sinus; 4 is zygomatic process of the temporal bone; 5 is articular disc; 6 is mandibular condyle; 7 is joint capsule; 8 is cortical bone; 9 is masseter muscle; 10 is ramus of mandible; 11 is lateral pterygoid muscle.

In this study, anatomical structures of the TMJ of the mature camel were identified and labelled in five figures (Figures 1, 2, 3, 4, and 5). 

### 3.1. CT Images (Figures 1 and 2)

The use of the soft-tissue window permits to identify the articular surfaces (articular cartilage and subchondral bone), articular disc and joint capsule, and the relationships between the TMJ, external acoustic meatus and masticatory muscles. Each of these soft tissues showed variable shades of grey, and the synovial fluid was the lowest attenuating structure. The squamous part of the temporal bone and the mandible were easily identifiable because of the high CT density in cortical bone and the intermediate CT density in their medullary cavities. The articular disc and joint capsule produced low to intermediate attenuation. It was not possible to identify the ligaments, blood vessels, and nerves in CT images. The post-processing of CT images allowed us to appreciate different bone structures and soft tissues of the TMJ, assisting in the interpretation of the CT images.

### 3.2. MRI (Figures 3 and 4)

Anatomical details of the camel TMJ were evaluated according to the characteristics of signal of the different tissues. 

On FSE T1-weighting sequences, the TMJ bony components showed high signal intensity with a granular appearance of the trabecular bone and bone marrow owing to fatty infiltration and no signal intensity (black) of the cortical and subchondral bone. The articular fibrocartilage disc and joint capsule produced low-to-intermediate signal intensity. The parotid salivary gland and masticatory muscles were identified. It was not possible to identify the TMJ ligaments, blood vessels, and nerves in CT images. 

On FSE T2-weighted MR images, the cortical and subchondral bone showed the same dark signal intensity, and the bone marrow appeared less bright than that on SE T1-weighted images. The articular fibrocartilage disc and joint capsule produced higher signal intensity. The parotid salivary gland and muscles had a intermediate signal intensity and appeared grey. Blood vessels were not differentiated because there was no blood flow as the camels were dead. 

The after processing of MR images allowed us to appreciate different bone structures and soft tissues of the TMJ, assisting in the interpretation of the MR images.

### 3.3. Transverse Anatomical Section of the Left TMJ (Figure 5)

The section used in this study provides the comparison with the CT and MR images and allowed the verification of anatomic details.

## 4. Discussion

To the authors' knowledge, there are no detailed published studies of the TMJ of the dromedary camel using CT and MRI, which turns out to be essential for morphologic, physiologic, and clinical studies involving bones and soft tissues located in this joint.

CT imaging is a cross-sectional diagnostic technique that provided excellent detail of clinically relevant anatomy and offers considerable advantages compared with traditional radiography and ultrasound for the examination of the TMJ: a lack of superimposition of the tissues and a higher differentiation of tissue densities [24, 25, 29]. CT provides excellent spatial resolution and good discrimination between bone and soft tissue [9–14, 24, 25, 29, 30]. CT is more sensitive in detecting diseases and distinguishes normal and abnormal structures accurately [22–24]. 

FSE T1- and T2-weighted MR images of the camel TMJ provided details of clinically relevant anatomy, and there were discrimination of both soft and mineralized tissues [15–21, 29]. MRI leads to an excellent spatial resolution and good discrimination between the bones and the cephalic soft tissues, in comparison with other conventional image-based techniques, due to a higher contrast resolution of the anatomic structures. MRI is good for understanding the morphology and the positions of the soft tissues [15–21, 29]. In addition, an important advantage is that MRI is a powerful technique for obtaining images on various anatomic planes without repositioning of the animal. Nonetheless, MRI also shows some disadvantages in comparison to other exploratory procedures. We emphasize that sedation is required if the animal is alive, the high cost of such equipment and the lack of antennas for use in medicine of the camels. This is the reason why many authors have resorted to the low intensity of the MRI units or collaboration agreements with hospitals in human medicine [15–21]. It is also necessary to stress that MRI is an image-based diagnostic technique that does not detect the contents of calcium in organic tissues, and, therefore, its application on studies of the osseous system is not recommended. 

In our study, we used an MRI unit of 1.5 T with a superconductor magnet, which enabled us to obtain high-definition tomographies. The physical parameters (TR, TE, matrix, etc.) which we applied to obtain MRI on the sagittal and transverse spatial planes can be used as an initial valid reference for this type of exploratory studies on the TMJ of the camel, especially for scientists who initiate themselves in the application of these modern image-based diagnostic techniques. 

MRI obtained in the sagittal plane allowed us a better evaluation of the topographic anatomic structures on the median plane of the TMJ, fundamentally the articular surfaces and articular disc, as well as the associated and topographically related structures. The anatomic relationships were appreciated most easily in the transverse planes. The anatomy section of the TMJ allows a correct morphologic assessment and topographic evaluation of anatomical structures, being useful tool for the identification of CT and MRI images. A thorough understanding of normal TMJ anatomy on CT and MR images is essential to optimize the diagnosis of TMJ disorders [16, 21, 25]. In the same way, we consider it quite useful to be able to establish some references on TMJ, in order to scan only selected parts during a clinical or experimental approach.

The use of CT and MRI in camels is limited because of cost, availability, and logistical problems to acquire imaging in large animals [9–11, 13, 14, 17, 19–25]. With developing technology [30], CT and MR imaging may soon become more readily available for diagnostic imaging in veterinary medicine.

## 5. Conclusions

Our study contribute to a better anatomical knowledge of the TMJ of the dromedary camel by means of CT and MRI and is a useful initial reference for clinical studies. CT is an excellent method for the detailed assessment of the bony structures. MRI is a valid imaging modality for the evaluation of the soft tissues. The FSE T1-weighted sequence should be the baseline to identify the anatomy, and FSE T2-weighted sequence will better enhance the study of the articular surfaces and articular disc. The after processing of CT and MR images allowed us to appreciate different bone structures and soft tissues of the TMJ, assisting in the interpretation of the images.
